# Soft-Tissue Analysis of Different Sagittal Skeletal Patterns Using the Geometric Morphometric Method

**DOI:** 10.1055/s-0042-1743149

**Published:** 2022-04-18

**Authors:** Tamana Sazgar, Nagham M. Al-Jaf, Noraina Hafizan Norman, Aspalilah Alias

**Affiliations:** 1 Centre of Pediatric Dentistry and Orthodontic Studies, Faculty of Dentistry, Universiti Teknologi MARA, Selangor, Malaysia; 2 Department of Orthodontics, Faculty of Dentistry, Kabul University of Medical Sciences, Kabul, Afghanistan; 3 Centre of Comprehensive Care Studies, Faculty of Dentistry, Universiti Teknologi MARA, Selangor, Malaysia; 4 Department of Basic Sciences and Oral Biology, Faculty of Dentistry, Universiti Sains Islam Malaysia, Kuala Lumpur, Malaysia

**Keywords:** Geometric morphometrics method, Soft-tissue analysis, Sagittal skeletal patterns, Shape variations

## Abstract

**Objectives** This study aimed to investigate the size and shape variations of soft-tissue patterns in different sagittal skeletal patterns using the geometric morphometrics method (GMM) obtained from lateral cephalograms.

**Materials and Methods** This is a retrospective study, where the sample comprised of 188 Malaysian Malay subjects aged between 18 and 40 years and with different sagittal skeletal patterns. Overall, 71 males and 117 females were gathered for all size and shape analyses. This study incorporated 11 soft-tissue landmarks, which underwent landmark application using tpsDig2 software version 2.31, while the shape analysis was done using MorphoJ software version 1.07a.

**Statistical Analysis** Statistical analyses were performed using IBM SPSS Statistics 26. The result of the analysis of variance (ANOVA) test showed significant differences in some of the parameters between the landmarks. Length D, Length E, Length F, Length H, and Length I showed significant differences ( *p < 0* .05), while other parameters showed no difference ( *p* > 0.05).

**Results** The shape variation of soft-tissue landmarks in different skeletal patterns existed in 18 different dimensions which showed by 18 principal components (PCs). Procrustes ANOVA and canonical variate analysis showed the size and shape differences of soft-tissue patterns between Class II and III and gender groups ( *p* < 0.0001). In discriminant function analysis for Class II subjects, the classification accuracy was 98.4%, whereas subsequent to cross-validation, the classification accuracy was 90.6%. For Class III subjects, the classification accuracy was 96.6%, while after cross-validation, the classification accuracy was 90%.

**Conclusion** Different sagittal skeletal patterns demonstrated different soft-tissue shape variations. Class III showed the most protrusive upper and lower lips, while Class II demonstrated the most retrusive lower lip.

## Introduction

One of the objectives of orthodontic treatment is an aesthetically pleasing and balanced face. An understanding of the soft tissues and their usual ranges allows for treatment planning to normalize the facial features of a given individual. 1 A detailed study of soft tissue is essential for orthodontic treatment and orthognathic surgery. Treatment based solely on the correction of hard-tissue values without taking into consideration the soft-tissue profile does not provide precise results. 2 The facial contours are formed by soft tissues, which can be altered by growth and orthodontic treatment. Orthodontists consider diagnosing and preparing the treatment plan with hard tissues, whereas the skeletal and dental relationships are the basis of soft tissue. However, a harmonious base does not indicate harmony and aesthetics of the overlying facial tissue. 3 The cephalometric skeletal and dental measurements have conventionally influenced treatment decisions in the orthodontic field. The recognition that the soft tissues may not closely match the underlying structures has shifted the focus of treatment planning toward soft-tissue evaluation; however, the extent of the interrelationship between soft and hard tissue is largely unknown. 4 For instance, the nasal profile shape and the upper lip contour are difficult to reconstruct or reliably predict in facial approximations; also, the soft-tissue shape might not follow the underlying structures as closely as estimated. 5 Dental and skeletal pattern analyses alone may be insufficient or deceptive due to marked differences in the soft tissues covering the dentoskeletal frame. Today, facial appearance is an important diagnostic criterion that should be considered in orthodontic treatment planning.

Facial profile harmony and balance vary among different types of facial and malocclusions of every ethnic group. The differences between various skeletal malocclusions can be seen in patients with orthodontic treatment or orthognathic surgery, both during diagnosis and treatment planning. 6

Comprehension of the facial morphology of different ethnic groups may aid in improved diagnosis and treatment planning for each race. To achieve treatment goals, soft-tissue values should match the norms of each ethnic population. For instance, to develop a database, the facial morphology of Colombian population was characterized using three-dimensional (3D) imaging and compared to the Caucasians. Significant ethnic differences in linear and angular measures were observed, thus emphasizing the need to consider soft-tissue findings when diagnosing and treating patients. 7 Two studies reported on the differences of facial features in 3D between Zimbabwe and the U.S. populations, and African American and Caucasian populations. Further to the above, the goal of this research is to create an average facial template for each population, allowing clinicians to treat patients based on their cultural aesthetic impressions. 8 9 In brief, each ethnic group has its unique morphologic norms, which is not applicable to other ethnic groups.

Cephalometry is a radiographic technique that was revolutionized in 1931 by Broadbent in the field of orthodontics. Researchers have applied cephalometric techniques to measure and record the changes in the jaw relative to the rest of the head. 10 Cephalometric radiography is a diagnostic approach that describes the growth and morphology of the craniofacial skeleton and is used for growth prediction, treatment planning, and assessment of treatment results. Most of these activities involve the identification of certain landmarks, as well as the calculation of different angular and linear variables. Among numerous limitations of the cephalometric analysis method, the errors can be classified into two categories: (1) errors of projection, and (2) errors of identification. Errors of projection are caused by the representation of a 3D to a 2D object. 11 12 Conventional cephalometric analysis cannot properly analyze craniofacial shape due to its inability to effectively reproduce the shape information provided by the cephalogram. One of the most important limitations of conventional cephalometric analysis is the absence of objectivity. Therefore, investigators may select which landmarks to record and variables to assess. 13

The recommended geometric morphometrics method (GMM) is managed to efficiently resolve unsolved difficulties in traditional cephalometric analysis. The use of GMM can overcome the shortcomings of cephalometric analysis, which can conduct both shape analysis and statistical analysis of objects. Geometric morphometrics is an essential new paradigm for the statistical studying of variation and covariation of the shapes of biological structures. The relative positions of points belonging to morphological landmarks are utilized as the shape concept. 14 Rohlf and Marcus 15 also pointed to the statement about the GMM that this new approach (GMM) allows to define, partition, and analyze the shape variations in populations of species, regardless of changes in scaling (size components). Geometric morphometrics precisely study the co-variation of shape components with their underlying causal factors, such as age, sex, and size. A basic property of GMM is that all landmarks are weighted equally, which ensures that the reference system is free of bias. Moreover, the shape of the craniofacial complex may be recorded and characterized in detail, rather than being exposed to the incomplete analysis of angles and ratios which is a shortcoming in conventional cephalometric. 16

Since the aim of orthodontic treatment is to achieve a harmonious relationship between the skeletal, dental, and soft tissue, the assessment of soft-tissue patterns besides hard-tissue patterns is another crucial point. The advent of advanced and useful GMMs has made it easy for orthodontists and maxillofacial surgeons to measure the shape variations of craniofacial structures in 2D and 3D that were not previously possible; presently, researchers are also focusing on the use of geometric morphometrics analysis. Therefore, this study characterizes soft-tissue landmarks in different sagittal skeletal patterns derived from lateral cephalogram based on the GMM.

## Materials and Methods

### Research Design

This retrospective study was carried out at the Faculty of Dentistry, Universiti Teknologi Mara (UiTM) and Faculty of Dentistry, Universiti Sains Islam Malaysia (USIM) to characterize the soft-tissue landmarks in different sagittal skeletal patterns in the Malay ethnic group. The data were collected from UiTM Dental Hospital and USIM Dental Clinic databases.

### Data Collection and Analysis

Inclusion criteria embraced Malay ethnic group patients, subjects aged between 18 and 40 years, patients with full permanent dentition (excluding the third molars), 17 and diagnostically acceptable lateral cephalograms. Exclusion criteria included patients with a history of orthognathic surgery, patients with craniofacial anomalies, history of orthodontic treatment, patients with mixed dentition, and non-Malay. The sample size for each type of sagittal skeletal pattern group was 64, and it was determined via G*Power calculation program version 3.0.10. The sample comprised 188 Malaysian Malay subjects aged between 18 and 40 years with different sagittal skeletal patterns. Overall, 71 males and 117 females were gathered for all size and shape analyses.

In the present study, the research tools were tpsDig2 software version 2.31 18 for landmark application and MorphoJ software version 1.07a 19 for shape analysis, while Notepad ^++^ version 7.8.2 and Microsoft office program Excel 2011 were used for data management. The first step in the GMM was the landmarks application. A total of 11 landmarks were applied to the lateral cephalograms of the patients using tpsDig2 software version 2.31. 18 All patients in this study must have the same number of landmarks, and the landmarks must be homologous, where each landmark must represent the same anatomical feature.

**Fig. 1:**
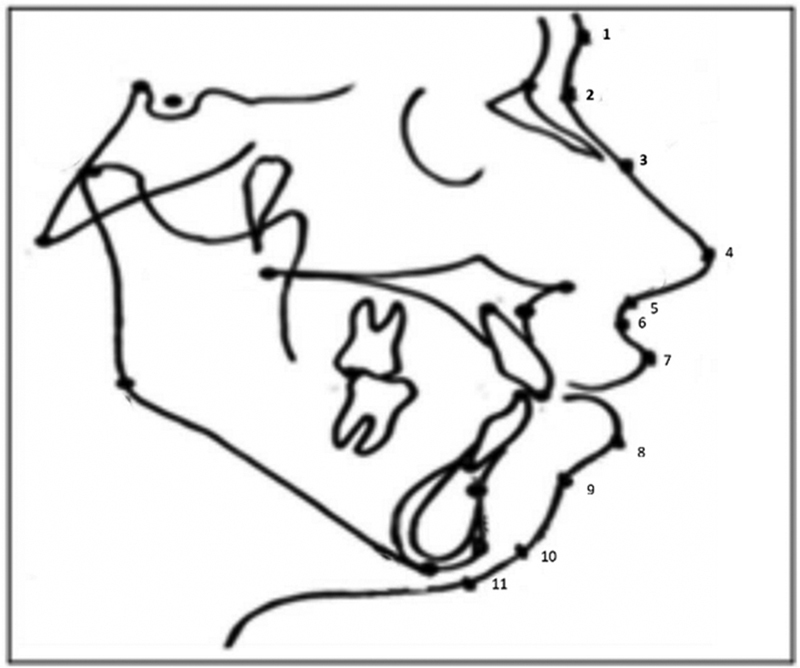
Map of the 11 soft tissue landmarks.

After landmarks digitizing, the data were exported from the Notepad file to Notepad ^++^ version 7.8.2, and raw coordinates of the data were collected using Notepad ^++^ version 7.8.2 prior to the shape analysis. Each specimen had 11 landmarks with x and y coordinates. The shape analysis software, namely MorphoJ software version 1.07a, 19 was used to analyze the 2D coordinates of the landmarks. To eliminate non-shape variations in the sample, raw landmark coordinates from all cephalograms were first analyzed using generalized Procrustes analysis. A series of principal component analyses (PCA) were used to explore the difference between samples in different types of malocclusions. Canonical variate analysis (CVA) was done to investigate the differences between the skeletal patterns and genders. Discriminant function analysis with cross-validation was used to assess the classification accuracy of skeletal patterns and genders. 20 A total of 11 2D soft-tissue landmarks were applied ( Fig. 1 ); these landmarks are defined in Table 1 . 21 Statistical analyses were performed using IBM SPSS Statistics 26 (IBM Corp, Armonk, New York, NY, United States). Data analysis was done to establish the mean and variance of nine soft-tissue parameters ( Table 2 ).

**Table 1:** Definition of soft-tissue landmarks

Number point	Landmarks	Definition
1	Soft-tissue glabella (G)	The most prominent or anterior point in the midsagittal plane of the forehead at the level of the superior orbital ridges.
2	Soft-tissue nasion (N)	The concave or retruded point in the soft-tissue overlying the area of the frontonasal suture.
3	Nasal crown (NC)	A point along the bridge of the nose halfway between soft-tissue nasion (N) and pronasale (Pn).
4	Pronasale (Pn)	The most prominent or anterior point of the nose.
5	Subnasale (Sn)	The point at which the nasal septum between the nostril's merges with the upper cutaneous tip in the midsagittal plane.
6	Subspinale (Ss)	The point of greatest concavity in the middle of the upper lip between subnasale (Sn) and labrale superius (Ls).
7	Labrale superius (Ls)	The most anterior point on the margin of the upper membranous lip.
8	Labrale inferius (Li)	The most anterior point on the lower margin of the lower membrane lip.
9	Soft-tissue submentale (B-point)	The point of greatest concavity in the middle of the lip between labrale inferius (Li) and soft-tissue pogonion (Pog).
10	Soft-tissue pogonion (Pog)	The most prominent or anterior point on the soft-tissue chin in the midsagittal plane.
11	Soft-tissue gnathion (Gn)	The midpoint between the most anterior and inferior points of the soft-tissue chin in the midsagittal plane.

**Table 2:** Description of soft-tissue parameters

Number	Parameters	Landmarks	Description
1	A	1–2	The distance between soft tissue glabella and soft-tissue nasion.
2	B	2–4	The distance between soft-tissue nasion and pronasale.
3	C	4–5	The distance between pronasale and subnasale.
4	D	5–6	The distance between subnasale and subspinale.
5	E	6–7	The distance between subspinale and labrale superius.
6	F	7–8	The distance between labrale superius and labrale inferius.
7	G	8–9	The distance between labrale inferius and soft-tissue submentale.
8	H	9–10	The distance between soft-tissue submentale and soft-tissue pogonion.
9	I	10–11	The distance between soft-tissue pogonion and soft-tissue gnathion.

## Results

### Shape Analysis

In this study, Principal component analysis (PCA) has displayed the major features of shape variation of soft-tissue patterns between Class I, Class II, and Class III sagittal skeletal patterns. The results of the PCA produced 18 PCs, which indicated that variances existed in 18 different dimensions in the data. The PC1 to PC5 showed significant differences among the 18 PCs and that cumulatively accounted for 80% of total shape variance. The first-three PCs were statistically meaningful as PC1 contributed to 38.644% of the total variance in the sample, while PC2 contributed to 20.976% of the total variance and PC3 contributed to 9.732% of the total variance ( Table 3 ).

**Table 3:** Percent variance and cumulative percent of soft-tissue pattern in different skeletal patterns

PC	Eigenvalues	Percent variance (%)	Cumulative variance (%)
**1**	0.00166487	38.644	38.644
2	0.00090372	20.976	59.620
3	0.00041930	9.732	69.352
4	0.00026595	6.173	75.525
5	0.00021942	5.093	80.618
6	0.00018243	4.234	84.853
7	0.00014999	3.481	88.334
8	0.00010851	2.519	90.853
9	0.00010020	2.326	93.179
10	0.00007608	1.766	94.945
11	0.00005618	1.304	96.249
12	0.00004684	1.087	97.336
13	0.00003454	0.802	98.138
14	0.00002637	0.612	98.750
15	0.00001953	0.453	99.203
16	0.00001493	0.347	99.549
17	0.00001017	0.236	99.785
18	0.00000925	0.215	100.000

**Fig. 2:**
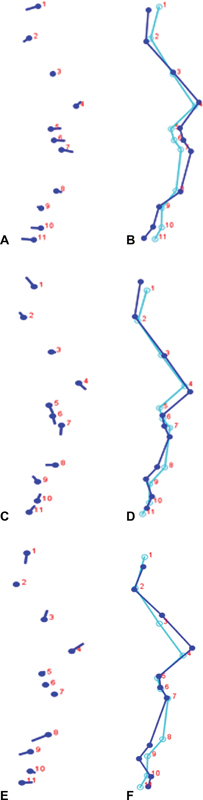
Lollipop and wireframe graphs of PC1, PC2, and PC3 shapes of soft-tissue pattern in skeletal patterns ( **A** and **B** ) PC1, ( **C** and **D** ) PC2, and ( **E** and **F** ) PC3.

The shape changes associated with PCs were observed in lollipop graphs and varying wireframe graphs for all 18 PCs. In Fig. 2 , the lollipop and wireframe graphs of PC1 to PC3 demonstrated alterations in their mean shapes and variations. All 11 soft-tissue landmarks displayed some level of variation from the mean. Soft-tissue glabella, subnasale, subspinale, labrale superius soft-tissue pogonion, and soft-tissue gnathion showed significant variances from the mean, while soft-tissue submentale, soft-tissue nasion, nasal crown, pronasale, and labrale inferius showed little or no variance in the specimens.

### Procrustes ANOVA of Soft-Tissue Pattern in Different Sagittal Skeletal Patterns

The Procrustes analysis of variance (ANOVA) evaluated the variation among individuals and error measurement in the specimens. The result of the Procrustes ANOVA analysis represented the different effects of skeletal patterns and gender groups that were demonstrated for centroid size and shape in separate ANOVA tables.

In this study, the gender groups showed significant differences in centroid size ( *p <* 0.05), while the skeletal patterns showed no difference ( *p* > 0.01). Goodall's F statistic ( *F* ) showed the lowest value in skeletal patterns ( *F* = 0.24), while the gender groups showed the highest *F* -value ( *F* = 6.51). The results showed that the size difference was significant in gender groups and not significant in skeletal patterns ( Table 4 ).

**Table 4:** Effect of size on the skeletal patterns and gender groups

Effect	SS	MS	D	F	*p*
Skeletal patterns	44745.342344	22372.671172	2	0.24	0.7883
Gender groups	589063.472018	589063.47201	1	6.51	0.0115 *

Abbreviations: DF, degree of freedom; MS, mean squares; SS, sum of squares.

* *p* < 0.05.

Procrustes ANOVA showed significant variation of shape for skeletal patterns and gender groups ( *p <* 0.0001). Goodall's F statistic ( *F* ) showed the highest value in skeletal patterns ( *F* = 25.71). The results showed that the shape has significant differences in skeletal patterns and gender groups. The Procrustes ANOVA established a highly significant morphological variation between skeletal patterns and gender groups ( *p <* 0.0001) ( Table 5 ).

**Table 5:** Effect of shape on the skeletal patterns and gender groups

Effect	SS	MS	DF	F	*p* -Value
Skeletal patterns	0.1752	0.00486	36	25.71	0.0001 **
Gender groups	0.0188	0.00104	18	4.47	0.0001 **

Abbreviations: DF, degree of freedom; MS, mean squares; SS, sum of squares.

** *p* < 0.001.

### CVA for Soft-Tissue Pattern between Class I, Class II, and Class III Sagittal Skeletal Patterns

**Fig. 3:**
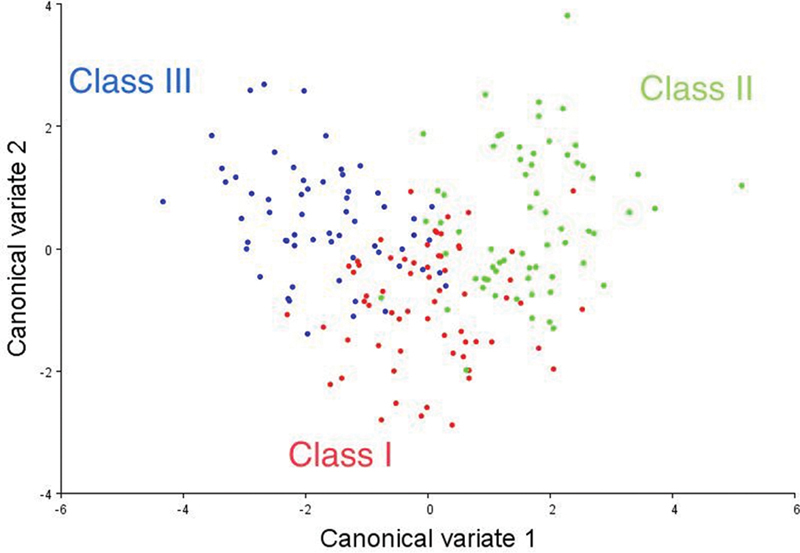
CVA of soft tissue in Class I, Class II, and Class III skeletal patterns. CVA, Canonical Variate Analysis.

CVA was conducted to investigate the soft-tissue pattern differences between Class I, Class II, and Class III skeletal patterns. There was a significant soft-tissue pattern difference between Class I, Class II, and Class III skeletal patterns in mean shape ( Fig. 3 ).

### Discriminant Function Analysis of Soft-Tissue Pattern between Class I, Class II, and Class III Sagittal Skeletal Patterns

In this study, discriminant function analysis (DFA) was carried out to explore the soft-tissue pattern shape differences between skeletal patterns and gender groups and to test the significance of the group differences. To evaluate the classification accuracy, cross-validation was used. All analyses were conducted separately.

### DFA between Sagittal Skeletal Patterns

The DFA with cross-validation was used to explore the soft-tissue pattern mean morphological differences between Class I, Class II, and Class III skeletal patterns. The DFA was done between classes [Class I with Class II], [Class I with Class III], [Class II with Class III], and gender groups. The classification accuracy and cross-validation percentages of skeletal patterns were calculated from the predicted group membership divided by the total number of samples in the group. The classification accuracy and cross-validation percentages among skeletal patterns were high between Class II and Class III.

**Fig. 4:**
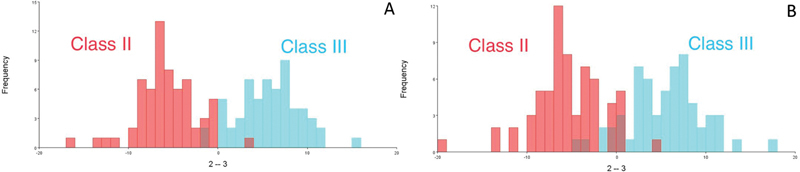
Discriminant function analysis graphs between Class II and III sagittal skeletal patterns. ( **A** ) Discriminant function score. ( **B** ) Cross-validation score.

For Class II subjects, the classification accuracy was 98.4%, while after cross-validation, the classification accuracy was 90.6%. The classification accuracy for Class III subjects was 96.6%, while after cross-validation, the classification accuracy was 90% ( Table 6 ). Fig. 4 shows the discriminant function test and after cross-validation test on MorphoJ software Version 1.07a 19 between Class II and Class III.

**Table 6:** Discriminant function test and cross-validation test on Class II and Class III

	Class II (cross-validation)	Class III (cross-validation)	Total	Classification accuracy (cross-validation) (%)
Class II	63(58)	1 (6)	64	98.4% (90.6%)
Class III	2(6)	58 (54)	60	96.6% (90%)

### DFA between Genders

**Fig. 5:**
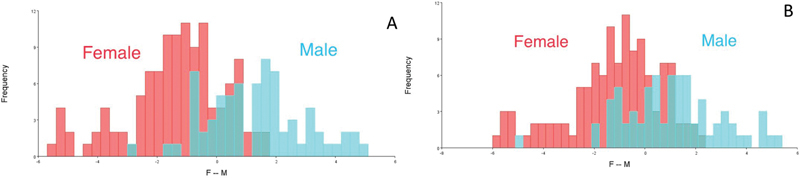
Discriminant function analysis graphs between females and males in Class I, II, and III sagittal skeletal patterns. ( **A** ) Discriminant function score. ( **B** ) Cross-validation score.

The DFA with cross-validation was used to explore the soft-tissue morphological differences of the facial shape change between female and male groups in Class I, Class II, and Class III skeletal patterns. For female subjects, the classification accuracy was 79.4%, while after cross-validation, the classification accuracy was 74.3%. The classification accuracy was 78.8% for male subjects, while after cross-validation, the classification accuracy was 73.2% ( Table 7 ). Fig. 5 shows the discriminant function test and after cross-validation test on MorphoJ software Version 1.07a 19 between the males and the females.

**Table 7:** Discriminant function test and cross-validation test on females and males

	Female (cross-validation)	Male (cross-validation)	Total	Classification accuracy (cross-validation) (%)
Female	93(87)	24 (30)	117	79.4% (74.3%)
Male	15(19)	56 (52)	71	78.8% (73.2%)

### Other Statistical Analysis

The result of the ANOVA test illustrated major variances in some of the parameters between the landmarks. Length D, Length E, Length F, Length H, and Length I showed significant differences ( *p < 0* .05), while other parameters showed no difference ( *p* > 0.05) ( Table 8 ).

**Table 8:** ANOVA test between parameters (linking of two landmarks)

		DF	Mean square	F	Sig.
Length A	Between groups	2	231.198	0.091	0.913
	Within groups	185	2531.843		
	Total	187			
Length B	Between groups	2	119.277	0.067	0.936
	Within groups	185	1792.208		
	Total	187			
Length C	Between groups	2	1998.469	0.501	0.607
	Within groups	185	3992.877		
	Total	187			
Length D	Between groups	2	449.243	3.440	0.034 a
	Within groups	185	130.613		
	Total	187			
Length E	Between groups	2	327.773	4.824	0.009 b
	Within groups	185	67.944		
	Total	187			
Length F	Between groups	2	19116.467	5.028	0.007 b
	Within groups	185	3801.816		
	Total	187			
Length G	Between groups	2	1119.117	0.555	0.575
	Within groups	185	2015.773		
	Total	187			
Length H	Between groups	2	11926.674	12.262	0.000 b
	Within groups	185	972.625		
	Total	187			
Length I	Between groups	2	2070.359	5.170	0.007 b
	Within groups	185	400.481		
	Total	187			

Abbreviation: ANOVA, analysis of variance.

a *p* < 0.05, significant difference.

b *p* < 0.01, significant differences.

## Discussion

Hard- and soft-tissue patterns with dentoskeletal diagnosis are essential for treatment planning in orthodontics and maxillofacial surgery. With the advancement of orthodontic treatment, the treatment of orthodontic cases is not restricted to correction of teeth, rather to achieve a good facial esthetic along with harmony and balance between dental-, hard-, and soft-tissue patterns. The advent of advanced and useful GMMs could assist the orthodontist in measuring the shape variations of soft-tissue patterns in 2D and 3D. Since the goals of orthodontic treatment are the harmonious relationship between the skeletal, dental-, soft-, and hard-tissue patterns. Moreover, all parts of the soft-tissue structure did not follow the underlying skeletal patterns 22 ; therefore, this study was established to evaluate the soft-tissue patterns in different sagittal skeletal patterns in Malaysian Malays using geometric morphometrics analysis.

The soft-tissue values are as important as hard-tissue values for the successful diagnosis and assessment of orthodontic treatment. Arnett & Bergman 23 stated that soft-tissue evaluation is essential in orthodontic treatment, and a complete understanding of facial features prior to treatment is needed; both hard and soft tissue must be considered to achieve an appropriate occlusion and harmonious facial aesthetics in orthodontic treatment. Holdaway 24 demonstrated that treatment systems that rely solely on hard-tissue measurements or reference lines might create unsatisfactory results.

The correlation between hard- and soft-tissue patterns is still under discussion; to what extent the skeletal structures affect the soft-tissue features and to what extent the hard- and soft-tissue patterns have correlation, different studies depicted different results. Kasai 25 stated that alterations in the underlying skeletal structure during orthodontic treatment are not directly reflected in the soft-tissue profile. Some components of the soft-tissue profile, such as the stomion and labrale inferius, have substantial connections with changes in the underlying skeletal structures, whereas other parts are less affected by changes in skeletal structures. Halazonetis 26 reported that significant correlations were found between the skeletal and the soft-tissue components of approximately 50%. In facial approximations, the shape of the nasal profile and the upper lip contour is difficult to rebuild or anticipate correctly, and the soft tissues may not always follow the underlying structures as closely as one may assume. In this study, the soft tissue patterns showed 18 different shape variances with the upper and lower lips showing the highest variations.

Zedníková Malá et al 5 in their study assessed to what extent the external facial shape was dedicated by skeletal and dental structures or by the soft tissues themselves. The mean predictive power of the entire craniofacial profile curve from the glabella to the menton (without the forehead) was 23.2%. This suggests that the variability of soft-tissue shape related to the shape of hard tissues accounted for 23.2%. At the same time, the other 76.8% could be ascribed to soft-tissue-specific factors. In this study, the soft-tissue patterns located at the midline of the face (soft-tissue glabella to soft-tissue gnathion) were evaluated which showed different variances in wireframe models ( Fig. 2 ), and also statistical differences were noted between landmarks as described in Table 8

In the present study, the shape and size analyses of soft-tissue patterns were conducted for all skeletal patterns (Class I, II, and III). In PCA, different skeletal patterns showed 18 PCs or variances ( Table 3 ), which indicated that variances existed in 18 different dimensions, while a previous study on the Malaysian population 27 based on GMM showed 14 PCs and due to the number and type of landmarks that were used, there were differences between this study result and their result. Woon et al 27 used nine hard-tissue landmarks, while this study used 11 soft-tissue landmarks. The shape variability was found in different skeletal patterns ( Fig. 2 ), and the result of Procrustes ANOVA showed that skeletal patterns had significant shape differences ( *p <* 0.0001), while there were no differences in centroid size ( *p* > 0.01). Since the GMM distinguished the size and shape and showed the shape of the objects regardless of size. The result of this study revealed that the difference between skeletal patterns is because of shape variability and not the size which resembled previous studies that also used the GMM on dental and skeletal patterns. 27 28 29

CVA in the present study showed the shape differences between different sagittal skeletal patterns. The shapes of different skeletal patterns were overlapping, as Class I was located between Class II and III. However, skeletal Class II and III had very negligible overlapping ( Fig. 3 ). In addition, in CVA, the highest Mahalanobis distances and Procrustes distances were exhibited by Class II and III among skeletal patterns and showed significant differences between Class II and III skeletal patterns ( *p <* 0.0001). These findings are in agreement with previous studies which evaluated dental and skeletal relations, which were conducted on Malaysian sample, 27 Chilean sample, 28 and Caucasian sample 29 ; therefore, this study findings support a separate and distinct soft-tissue patterns shape for different patterns and relations especially for Class II and III with a negligible shape similarity.

Since in this study, the skeletal Class II and III shapes were different and had very negligible shapes overlapping in CVA. Moreover, previous different studies also reported significant differences between Class II and III patterns. For instance, differences between Class II and Class III skeletal relations, when the shape and size of the sella turcica in Saudi subjects were compared in different skeletal patterns, there were significant differences in the diameter of sella between the Class II and Class III subjects. Skeletal Class III had the larger diameter, while Class II subjects had the smaller diameter sizes. 30 Skeletal Class II and III showed significant differences in mandibular symphysis morphology. Class III skeletal group showed greater vertical symphysis dimension, chin length, and symphysis convexity, while skeletal Class II subjects had greater skeletal symphysis and dentoalveolar lengths. 31 The above-mentioned studies had a similarity and great agreement with this study result regarding differences between Class II and Class III skeletal relations in different skeletal landmarks and structures.

In this study, discriminant function analysis of soft-tissue pattern in different sagittal skeletal patterns showed the most classification accuracy for Class II and Class III skeletal relations, with success rates of 98.4, and 90.6% and after cross-validation, 96.6 and 90%, respectively. Previous studies also similarly showed different discriminant function scores, for instance, according to discriminant analysis, patients with early Class III malocclusion had a high degree of accurate classifications (93.3%). 32 The Class II Division 1 malocclusion classification accuracy in the discriminant analysis was 83% when comparison was made on the effects of bonded Herbst appliance in Class II Division 1 malocclusion. 33 The evaluation of mandibular changes treated with functional jaw orthopaedics is followed by fixed appliances in Class II patients, which showed 80% classification power in discriminant analysis. 34 Discriminant function analysis is usually used in studies for classification accuracy demonstration, where the abovementioned studies demonstrated different classification accuracy, which may be attributable to the different landmarks and variables used in different studies.

## Conclusion

Different sagittal skeletal patterns showed different soft-tissue shape variations.

- Class III showed the most protrusive upper and lower lips.
- Class II showed the most retrusive lower lip.

The results of our study can be used to develop diagnostic software of wireframe graphs for soft-tissue landmarks in different sagittal skeletal patterns which can assist orthodontists and maxillofacial surgeons in achieving a more successful and stable treatment result.
